# Summary of recent results obtained by the Sileye-3/Alteino detector in the Russian part of the International Space Station as part of the ALTCRISS project

**DOI:** 10.1093/jrr/rrt221

**Published:** 2014-03

**Authors:** Oscar Larsson, Viktor Benghin, Marco Casolino, Inna Chernikch, Luca Di Fino, Christer Fuglesang, Marianna Larosa, Bengt Lund-Jensen, Livio Narici, Viktor Nikolaev, Vladislav Petrov, Piergiorgio Picozza, Cristian De Santis, Veronica Zaconte

**Affiliations:** 1 Royal Institute of Technology, Stockholm, Sweden; 2 Institute for Biomedical Problems, Moscow, Russia; 3 INFN Sect. Roma Tor Vergata, Rome, Italy; 4 RIKEN, Saitama, Japan; 5 University of Rome Tor Vergata, Rome, Italy

**Keywords:** LET, ISS, Sileye3, Alteino, Altcriss, ALTEA

## Abstract

The Sileye3/Alteino experiment is devoted to the investigation of the light flash phenomenon and particle composition of the cosmic ray spectrum inside the ISS. The particle detector is a silicon telescope consisting of eight planes, each divided into 32 strips. Data acquisition was initiated in 2002 in the Russian Pirs module. The data on nuclei from C to Fe in the energy range above about 60 MeV/n presented here were taken as part of the ESA Altcriss project [ 1] from late 2005 through 2007. Here we report on LET, from different locations and orientations, in both the Pirs and Zvezda modules. Taking solar modulation into account the results are in agreement with ALTEA measurements from USLab [ 2]. To convert the energy deposition in Si to the equivalent in water, the logarithmic relation between LET in Si and water adopted from [ 3]. In Fig. 1, the LET spectra in water for Alteino and ALTEA are compared with DOSTEL spectrum from 2001 [ 4], and we see a good overall agreement. We are currently in the process of preparing a detailed paper on the dose and dose equivalent rates in different places inside the Zvezda and Pirs modules and a novel analysis of the contribution to the different doses as a function of strip hit multiplicity.

**Fig. 1.**
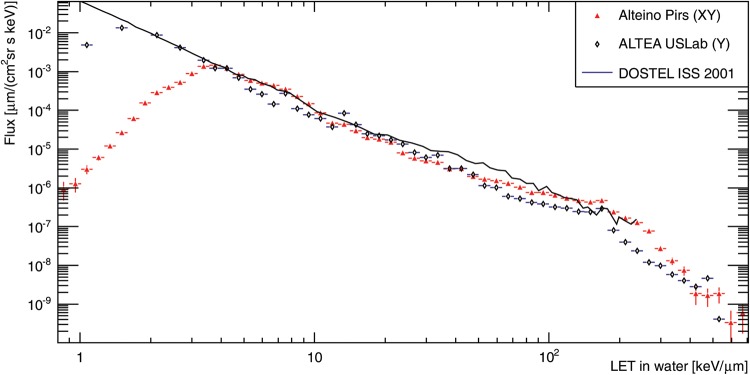
LET spectra in water from Alteino (red triangle), ALTEA (black diamond) [ 2], DOSTEL 2001 (solid line) [ 4].

LET spectra in water from Alteino (red triangle), ALTEA (black diamond) [ 2], DOSTEL 2001 (solid line) [ 4].
